# Characterizing Episodes of Lucidity in Dementia: Observational and Applied Computational Linguistics Approaches

**DOI:** 10.1093/geroni/igab046.179

**Published:** 2021-12-17

**Authors:** Andrea Gilmore Bykovskyi, Kim Mueller, Nicole Werner, Erica Smith, Laura Block, Clark Benson

**Affiliations:** 1 University of Wisconsin-Madison, Madison, Wisconsin, United States; 2 Industrial and Systems Engineering, Madison, Wisconsin, United States

## Abstract

Though episodes of lucidity (EL) in Alzheimer’s disease and related dementia (ADRD), reportedly more common near end of life, have significant implications for care, they are poorly understood due to underdeveloped methodological approaches for capturing and measuring these events. This prospective observational study addresses these gaps through audiovisual observation among persons with ADRD surrounding end of life to inform data-driven definitions for EL and distinguish EL from routine fluctuations in ADRD. Audiovisual observation is well-suited to addressing gaps in operationalization of EL, providing an objective data source to assess verbal and nonverbal communication, the primary means through which EL are evidenced. Our study is designed to establish optimal procedures for capturing audiovisual data of targeted populations and timeframes to maximize opportunities for detecting EL. Operationalization of EL will be informed by computational linguistic and behavioral-event coding of linguistic and non-linguistic communication features of EL and associated temporal qualities.

